# Determinants of aggregate length of hospital stay in the last year of life in Switzerland

**DOI:** 10.1186/s12913-016-1725-7

**Published:** 2016-09-01

**Authors:** Damian Hedinger, Julia Braun, Vladimir Kaplan, Matthias Bopp, for the Swiss National Cohort Study Group

**Affiliations:** 1 Epidemiology, Biostatistics and Prevention Institute, University of Zurich, Hirschengraben 84, CH-8001 Zürich, Switzerland; 2 Hospital of Muri, Muri, Switzerland

**Keywords:** Inpatient care, Length of hospital stay, End-of-life, Socio-demographic determinants, ICU, Regional variation

## Abstract

**Background:**

In contrast to individual preferences, most people in developed countries die in health care institutions, with a considerable impact on health care resource use and costs. However, evidence about determinants of aggregate length of hospital stay in the last year preceding death is scant.

**Methods:**

Nationwide individual patient data from Swiss hospital discharge statistics were linked with census and mortality records from the Swiss National Cohort. We explored determinants of aggregate length of hospital stay in the last year of life in *N* = 35,598 inpatients ≥65 years who deceased in 2007 or 2008.

**Results:**

The average aggregate length of hospital stay in the last year of life was substantially longer in the German speaking region compared to the French (IRR 1.36 [95 % CI 1.32–1.40]) and Italian (IRR 1.22 [95 % CI 1.16–1.29]) speaking region of the country. Increasing age, female sex, multimorbidity, being divorced, foreign nationality, and high educational level prolonged, whereas home ownership shortened the aggregate length of hospital stay. Individuals with complementary private health insurance plans had longer stays than those with compulsory health insurance plans (IRR 1.04 [95 % CI 1.01–1.07]).

**Conclusions:**

The aggregate length of hospital stay during the last year of life was substantially determined by regional and socio-demographic characteristics, and only partially explained by differential health conditions. Therefore, more detailed studies need to evaluate, whether these differences are based on patients’ health care needs and preferences, or whether they are supply-driven.

## Background

One of the major concerns relating to demographic aging is the increasing use of health care resources by the growing number of elderly (≥65 years) and oldest-old (≥80 years) patients. Survival and longevity have increased in several chronic conditions due to modern medical care [1]. However, there is evidence that chronic medical conditions and their multiples (multimorbidity) increase with age and before death [2]. Nowadays, the majority of people in industrialised countries die in health care institutions such as hospitals and nursing homes. However, there is a large variation in the proportion of deaths occurring in hospitals [3]. In Europe, these differences are often explained by the differing availability of alternative health care services for elderly and oldest-old patients (i.e., nursing homes) [4].

Previous research related to health care resource use has shown that the average cost of hospital care significantly increased with age. However, it is argued that not age per se is the main driver of health care costs, but ultimate closeness to death [5, 6]. Nevertheless, the place of death has a considerable impact on health care resource use, since end-of-life care is generally more expensive in hospitals than in nursing homes, and even much more expensive than outpatient care at home [7, 8]. Studies from the US and the Netherlands have shown that the last-year-of-life health expenditures constituted between 10 and 25 % of all medical expenses during life [6, 8, 9]. There is evidence that these costs may even increase in the future.[10]. In Switzerland, health insurance has been compulsory since 1996 and covers, in principle, all medical treatments and diagnostics prescribed by doctors. It also covers the costs of medical care provided in long-term care institutions [11]. Patients contribute to the costs of care through co-insurance rates up to an annual ceiling and a modest flat daily co-payment for hospital stays. Three out of ten people have a private supplementary health insurance typically covering private rooms in hospitals. Switzerland-specific information concerning the costs of hospital stays in the last year of life is lacking. There is, however, no doubt that hospital inpatient stays are more expensive than any other kind of health care and therefore offer a large potential for cost saving.

A difficult clinical challenge is the admission of oldest-old patients to an intensive care unit (ICU). ICU care is expensive, highly rationed, morally charged, and therefore, decisions regarding admissions to ICUs remain crucial questions in oldest-old patients [12]. There is evidence that admission rates to acute care hospitals as place of death decreased, while ICU admissions in the last month of life increased [13]. In addition, huge differences regarding ICU admission rates and regarding physicians’ opinions of appropriateness of ICU care were observed between hospitals [13, 14]. Furthermore, adequate support for shared decision-making and patient-care specific measures can increase the family satisfaction with ICU care and therefore better meet the patient’s and his/her family’s preferences of end-of-life care [15].

It is well known that health care use depends not only on age and proximity to death, but also on other socio-demographic factors like gender, educational level, and marital status [16]. Educational differences regarding morbidity and mortality are an uncontested finding in social epidemiology [17–19]. There is also evidence that socio-demographic determinants have a significant influence on the place of death [20, 21]. Furthermore, there is solid evidence of regional differences in health care use and place of death [7, 22, 23]. However, to our knowledge, evidence on determinants of frequency and length of hospital stay in the time period preceding death is sparse.

We therefore explored, based on linked census and hospital discharge statistics, the potential impact of medical (e.g. multimorbidity) and social determinants (e.g., education, home ownership, marital status) on the aggregate length of hospital stay in the last year of life among those deceased in hospitals.

## Methods

### Data

We extracted data from two different sources covering all individuals living in Switzerland:

1. The Swiss National Cohort (SNC, www.swissnationalcohort.ch) is an anonymous linkage of census, mortality and emigration records [24]. We used individual data from the 2000 census and mortality data (incl. cause of death) from 2007-08.
2. The Swiss hospital discharge statistics (MedStat) administered by the Swiss Federal Statistical Office [25] encompasses individual information about diagnoses, treatments, discharge dates, and – crucial for record linkage – for those deceased in hospitals, dates of birth. We used data for the year 2002–2008.

Both data sources encompass the entire Swiss population and are fully anonymized. For reasons of data protection and confidentiality, there is no personal identifier that allows direct linkage of data sources on an individual level. The linkage had to rely on common identification variables such as place of residence, date of birth, and date of death. As a consequence, the linkage to the SNC was possible only for those who died in an institution. The detailed linkage process is described elsewhere [20]. We restricted our analyses to individuals ≥65 years (i.e., born before 1942) who died in 2007 and 2008 in hospital settings. As we know from a place of death study in Switzerland [26], about 45 % of men and 35 % of women above 65 years die in hospitals. In the study period, almost 99 % of deaths in hospitals could be linked with the SNC and are therefore accounted for in this analysis.

### Study design

**Fig. 1:**
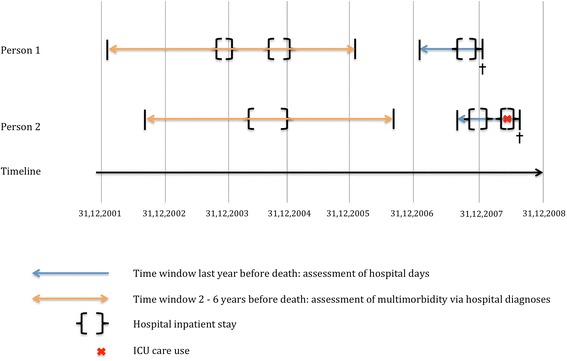
Examples of two persons with the corresponding time windows of different health variables

Our outcome variable was the aggregate number of days spent in a hospital during the last year (365 days) of life. The independent variables were grouped into medical, individual, familial/housing, and structural/regional attributes. As control variables we included age (at the time of death) and sex. Cause of death was categorized as: malignant neoplasms (ICD 10: C00-C99), coronary heart disease (I20-I25), stroke (I60-I69), chronic obstructive pulmonary disease (COPD, J40-J47), dementia (F01, F03, G30), and all other causes. From MEDSTAT, we derived information about multimorbidity (≥2 chronic conditions), assessed from inpatient diagnoses within 2–6 years before death. We defined chronic conditions using ICPC-2, of which 129 rubrics were classified as chronic conditions [27]. We used specific time windows in order to test the different impact of health indicators which are closer to or more distant from death. For better understanding of our health variables, Fig. 1 presents examples of two persons with corresponding time windows. In addition, we generated an ICU variable to identify the influence of ICU care during the last hospital stay: no ICU care, ICU care 1–48 h, or more than 48 h. From the 2000 census data, we derived the educational level according to the International Standard Classification of Education (ISCED), version 1997: no or low secondary education (ISCED 0–2), post-secondary non-tertiary (“medium”, ISCED 3–4), and tertiary education (“high”, ISCED 5). From the 2000 census we extracted information about home ownership (owner-occupier household). From MEDSTAT, we extracted the hospital room category of the respective hospitalisation (private/semi-private or regular). As very few patients upgrade their hospital room category from regular to private/semi-private, since they have to reimburse additional costs, the hospital room category is representative for the health insurance plan (private/compulsory). Marital status was derived from the SNC and assessed at the time of death (never married, married, widowed and divorced). Place of residence was categorized into the three main language areas in Switzerland, namely the German, French and Italian speaking part. The nationality was derived from the SNC and categorized as Swiss or foreign. To account for geographical variation in nursing home bed availability, we included the nursing home bed density per 100 inhabitants 65 years or older derived from the SNC.

### Statistical methods

For descriptive analyses, we calculated means, frequencies and proportions of the respective variables. To detect a right-skewed distribution, we report means, medians, SD and 25^th^ and 75^th^ percentiles of our outcomes. The outcome variable “aggregate number of days spent in a hospital in the last year of life” is a count variable. Therefore, a count regression such as Poisson or negative binomial is appropriate for statistical modelling. We chose the negative binomial model over the Poisson model to account for the strong over dispersion in the outcome variable. We also considered to use a zero-inflated negative binomial model. However, the number of zeros in our data set is not extremely large (less than 5 % of the study population) and there is no reason to believe that part of the patients differs from the others in that they per se cannot have any counts but zero. Goodness-of-fit criteria like AIC and BIC performed slightly worse in the zero-inflated model than in the regular binomial model and also the Vuong test showed a non-significant result with a p-value close to 1. For these reasons, we preferred a regular negative binomial model. We calculated incident rate ratios (IRR) [28] to assess the impact of several independent variables on the number of days spent in a hospital in the last year of life. For better understanding of the interpretation of IRR, we give a reading example using the estimated IRR of 1.05 for a person with “high” educational level (cf. Table 2): Compared to a person with “medium” educational level (reference category), the expected aggregate number of days spent in a hospital during the last year of life was for a person with “high” educational level 1.05 times as high as in the reference category. We used likelihood ratio tests to calculate joint p-values of categorical variables. The level of significance was set to α = 0.05 (two-sided). To improve the fit of the model, we explored relevant interaction effects between sex and marital status, cause of death and ICU use, and multimorbidity and ICU use. However, none of these interaction terms improved the model with respect to the Bayesian information criterion (BIC), and for this reason, they were not included in the final model. Due to substantial differences between the language regions, we performed also a stratified analysis for the two major language regions (German vs. French speaking part) of Switzerland. In addition, we also checked for the above mentioned interaction effects in each of the models, but again, with respect to the BIC, the interaction terms didn’t improve the models.

## Results

We identified *N* = 35,598 patients (18,993 men and 16,605 women) ≥65 years who died in 2007 and 2008 in hospital settings. Table 1 presents the characteristics of our study population.

**Table 1:** Baseline characteristics of patients deceased in hospitals in 2007 and 2008 (*N* = 35,598)

*Aggregate length of hospital stay in the last year of life (days)*	
Length of hospital stays last 365 days, mean	32.1
Length of hospital stays last 365 days, median	20
Length of hospital stays last 365 days, 25^th^ percentile	7
Length of hospital stays last 365 days, 75^th^ percentile	44
Length of hospital stays last 365 days, SD	38
*Mean age* at time of death (years)	80.8
*Sex*	
men	53.4 %
women	46.6 %
*Cause of death*	
cancer	35.5 %
coronary heart disease	12.5 %
stroke	8.0 %
chronic obstructive pulmonary disease	3.3 %
dementia	1.3 %
other	39.4 %
*Multimorbidity (assessed in time window 2–6 years before death)*	
no	23.2 %
yes	34.2 %
unknown (no hospital admission)	42.7 %
*Intensive care during last hospital stay (ICU)*	
none	83.2 %
1–48 h	8.2 %
more than 48 h	8.6 %
*Educational level*	
low	38.3 %
medium	34.9 %
high	11.3 %
unknown	15.6 %
*House ownership*	
owner-occupier	43.2 %
tenant	56.8 %
*Room category*	
regular	77.9 %
semi private/private	22.1 %
*Marital status*	
never married	8.0 %
married	50.4 %
widowed	34.5 %
divorced	7.1 %
*Nationality*	
Swiss	92.2 %
foreign	7.8 %
*Language region*	
German	67.5 %
French	27.4 %
Italian	5.1 %
Nursing home bed density*	6.7

* = average number of nursing home beds per 100 habitants aged 65 years and older (per 106 regions)

Data source: Swiss Federal Statistical Office, MedStat, SNC

We observed a strong right-skewed deviation of the distribution of aggregate number of days spent in a hospital during the last year of life. Less than one out of five patients received ICU care during the terminal hospital stay.

Aggregate length of hospital stay during the last year of life (mean, SD) and estimated incidence rate ratios (IRR) for baseline characteristics are presented in Table 2. A stratified analysis for the German and French speaking part of the country is given in Appendix 1 and 2.

**Table 2:** Mean aggregate length and results of the negative binomial regression analysis (*N* = 35,598)

	Length of stay (days)		IRR	*95 % CI*
	Mean	SD		
Age (at time of death) (*p* < 0.001)			0.98	*0.98–0.99*
Sex (*p* < 0.05)				
men	32.3	38.7	*1.00*	
women	31.9	38.2	1.03	*1.00–1.06*
Cause of death (*p* < 0.001)				
*cancer (ref.)*	39.1	36.4	*1.00*	
coronary heart disease	23.6	34.8	0.68	*0.65–0.70*
stroke	23.2	35.6	0.65	*0.62–0.68*
COPD	35.6	44.3	0.89	*0.83–0.95*
dementia	61.2	72.6	1.74	*1.57–1.92*
other	29.0	38.3	0.81	*0.78–0.83*
Multimorbidity (*p* < 0.001)				
*no (ref.)*	33.9	39.7	*1.00*	
yes	36.6	41.3	1.07	*1.04–1.10*
unknown (no hospital stay)	27.5	34.8	0.79	*0.77–0.82*
ICU during last stay (*p* < 0.001)				
*no (ref.)*	33.3	*39.5*	*1.00*	
1–48 h	20.3	29.9	0.65	*0.62–0.67*
more than 48 h	31.3	33.2	0.97	*0.93–1.01*
Educational level (*p* < 0.001)				
*medium (ref.)*	31.3	36.1	*1.00*	
low	31.3	37.6	0.98	*0.95–1.01*
high	34.4	41.5	1.05	*1.01–1.09*
unknown	34.0	43.2	1.06	*1.02–1.10*
House or flat owner (*p* < 0.001)				
*tenant (ref.)*	33.1	40.0	*1.00*	
owner-occupier	30.7	36.4	0.93	*0.91–0.95*
Room category (*p* < 0.01)				
*regular (ref.)*	32.4	*40.1*	*1.00*	
semi-private/private	31.1	32.2	1.04	*1.01–1.07*
Marital status (*p* < 0.001)				
*married (ref.)*	32.4	37.1	*1.00*	
never married	33.1	42.9	1.04	*0.99–1.08*
widowed	30.0	37.9	1.03	*1.00–1.06*
divorced	38.4	44.9	1.12	*1.07–1.17*
Nationality (*p* < 0.001)				
*Swiss (ref.)*	31.4	37.8	*1.00*	
foreign	39.8	45.1	1.09	*1.04–1.14*
Language region (*p* < 0.001)				
*German (ref.)*	27.6	34.1	*1.00*	
French	42.1	46.5	1.36	*1.32–1.40*
Italian	37.3	34.8	1.22	*1.16–1.29*
Nursing home bed density^a^ (*p* > 0.001)			0.96	*0.95–0.97*

*IRR* incidence rate ratios,  95 % CI = 95 % confidence interval, *p*-values from likelihood ratio tests

^a^ = average number of nursing home beds per 100 habitants aged 65 years and older (per 106 regions)

Data source: Swiss Federal Statistical Office, MedStat, SNC

Increasing age was a significant predictor for shorter aggregate length of hospital stay in the last year of life. There were also significant differences in aggregate length of stay with respect to specific causes of death: compared to patients dying of cancer, those dying of coronary heart disease, stroke, and chronic obstructive pulmonary disease had a significantly shorter, while those dying of dementia significantly longer length of hospital stay. Multimorbidity significantly increased the aggregate duration of hospital stay. For those without any hospital admission within 2–6 years before death (and consequently no information about multimorbidity), the aggregate time spent in hospitals during the last year of life was significantly shorter than for those previously hospitalized but without a diagnosis of multimorbidity. For those who received ICU care for ≤48 h during the last hospitalisation, aggregate length of stay during the last year of life was significantly shorter than for those who received no ICU care or ICU care for >48 h. There was a remarkable difference between the French and the German speaking part regarding those who received ICU for >48 h (Appendix Tables 1 and 2): While this group had a significantly longer mean aggregate length of stay in the German speaking part, we found the opposite effect in the French speaking part. Patients with a high or unknown educational level had significantly longer aggregate stays than those with a medium or low educational level. Home ownership was a significant predictor of shorter aggregate length of stay. Those hospitalized in a semi-private/private room category (proxy for a private health insurance plan) had a significant longer aggregate stay than those hospitalized in a regular room category (proxy for a compulsory health insurance plan). Divorced patients had significantly longer aggregate stays compared to those married, widowed, and never married. Swiss citizens had significantly shorter aggregate stays than foreigners. Compared to the German speaking region, the aggregate length of hospital stay in the last year of life was longer in the Italian and even more so in the French speaking part of Switzerland. We also found a significant effect of nursing bed density on the aggregate length of stay: high regional nursing bed density was associated with a shorter aggregate length of hospital stays during the last year of life.

## Discussion

As expected, health-related characteristics such as specific cause of death, multimorbidity, and admission to intensive care during the last hospitalization had a significant impact on the aggregate length of hospital stays during the last year of life. However, the duration was not dependent on health-related characteristics alone, but also on a variety of social determinants such as educational level, home ownership, hospital room category (proxy of the health insurance plan), language region, and nursing bed density.

A noteworthy observation was the substantial variation between aggregate length of hospital stays during the last year of life in the different language regions of Switzerland. Patients living in the Italian, and particularly those living in the French part spent more days in hospitals during the last year of life than those living in the German part. Prior studies gave already evidence for regional variation in health care use in Switzerland and in the US [7, 22]. Consistently, a recent study from Switzerland found that people living in the French or Italian part were more likely to die in an institution than those living in the German part [20]. One could argue that this might point to a higher propensity for more aggressive (and possibly futile) care at the end-of-life due to cultural differences between the language regions [29].

The well-known divergence between the preferred and actual place of death might be due to unmet wishes of patients and their families regarding hospital and long-term care admissions before death and might represent an indicator of a low quality of dying [30–32]. Therefore, home-based palliative care models, which decrease the time spent in hospitals during the last months of life, may increase the quality of dying and end-of-life care [33, 34]. Different palliative care models have been developed in some European countries to implement alternative ways and increase the quality of end-of-life care, however, their effectiveness and impact on care before death varies substantially [35]. A successful integration of palliative care services in the ICU can increase the quality, save costs and improve patient and family satisfaction [36]. Unfortunately, due to regional variation and lack of reliable data, an evaluation of palliative care in Switzerland is difficult [37].

Another remarkable observation is the variation of ICU care during the terminal hospitalization before death. In the US, around 20 % of deaths occur in ICU settings [9] despite questionable benefits regarding survival and quality of life [14]. Therefore, ICU care offers an important potential for health costs saving [9]. In our study population, 16.8 % of all patients received ICU care during the terminal hospitalization. Many of those with an ICU stay ≤48 h died shortly after admission. Such a “sudden death” was associated with a significant shorter length of aggregate hospital stay in the last year of life. However, regarding aggregate length of hospital stay in the last year of life, those with an ICU stay >48 h did not significantly differ from those receiving no ICU care at all.

Multimorbidity was another informative determinant of aggregate length of hospital stay during the last year of life: Multimorbidity diagnosed during hospitalizations within 2–6 years before death was associated with a longer aggregate length of hospital stay in the last year of life, probably due to worse health status and therefore more medical needs of these often very sick patients. Conversely, patients without hospitalization within 2–6 years before death (and therefore no information on multimorbidity) had substantially shorter aggregate hospital stays and received less medical care.

We also found evidence for effects of socio-economic differences on the aggregate length of hospital stays. However, compared to other studies on morbidity and mortality differences in the old age [17–19], our results were less consistent. Higher educational level was associated with increased aggregate length of stay. Conversely, home ownership, another proxy for higher socio-economic position, was associated with a significantly shorter aggregate length of stays. Patients with presumably better health insurance plans (semi-private/private room category) had longer stays compared to those with regular coverage plans. A similar study from Finland showed that educational level had little effect on hospital care in the last 7 years preceding death [16]. Conversely, socio-economic characteristics were important determinants of admission to and death in a nursing home but not of acute care hospital length of stay [16, 20]. Finally, regional attributes like language region and nursing home bed density were more consistent predictors of aggregate length of hospital stays in the last year of life compared to socio-economic determinants. As research on differences in health care between the two major language regions in Switzerland is scarce, it is difficult to elucidate our results. We found evidence for cultural differences in hospital treatments before death, because longer hospital stays, which imply more aggressive treatments before death, were more common in the French and Italian speaking part of Switzerland compared to the German speaking part. This result is in line with higher odds of hospitals as place of death in those regions [26]. On the other side, nursing home bed density – known to be lower in the French speaking part [38] – had a higher negative impact on length of hospital stays in this region, suggesting an important role of supply-sensitive care.

### Strengths and limitations

One major strength of our study is the national coverage and the size of the study population. Another strength is the uniqueness of the data base generated by linking census with mortality and other administrative data. Furthermore, our study gives empirical evidence for several health indicators considering an extended time period before, and therefore little related to death.

Besides such strengths, the study has some limitations: As usual for secondary data and particularly administrative data analyses, by far not all desirable information is available (e.g., information about palliative care) and the use of proxy variables with limited validity is inevitable. However, the use of such weak measures does not necessarily lead to a systematic information bias but rather results in a non-differential misclassification and therefore underestimation of the true association. In Switzerland, there is no statistical information on the outpatient health care. Therefore it was not possible to assess diagnostic information of people that have not been hospitalized during the study period. As we restricted the study population to people having died in hospitals, our study population is not representative for the general population above 65 years. This is the reason why some causes of death (e.g. dementia) have only small percentages. Another limitation of the study is the incomplete linkage of all deaths to a census record as well as to a respective hospital record.

The percentage of hospital deaths is in line with another study from Switzerland [39]. Our study is based on a virtually complete nationwide assessment of hospitalizations in 2002–2008 and thus mirrors the situation in Switzerland at that time. But due to differences in methodology, study populations and health care systems, we cannot validly compare our results with those of other countries.

## Conclusions

The aggregate time spent in hospitals in the last year of life is mainly determined by differential health conditions and therefore, differential needs of medical care. However, remarkable unexplained disparities between the German and the Latin (French and Italian) speaking region of Switzerland remain: frequent and/or longer hospital stays in the last year of life are more common in the French and Italian speaking part of the country, probably due to cultural differences. Therefore, more detailed studies need to evaluate, whether these differences are based on patients’ health care needs and preferences, or whether they are supply-driven. As hospital care in general and ICU care in particular is expensive and often unwanted by those deceasing, more efforts to minimize aggressive care at the end-of-life – especially in the Latin regions of Switzerland – may be a promising target of health policy.

## Appendix

**Table 3:** Results for the German speaking part of Switzerland (*N* = 24,041)

	Length of stay (days)		IRR	*95 % CI*
	Mean	SD		
Age (at time of death) (*p* < 0.001)			0.98	*0.98–0.98*
Sex (*p* < 0.05)				
men	27.9	35.0	*1.00*	
women	27.4	33.8	1.04	*1.00–1.07*
Cause of death (*p* < 0.001)				
*cancer (ref.)*	34.1	32.5	*1.00*	
coronary heart disease	21.4	31.8	0.68	*0.65–0.72*
stroke	19.6	30.4	0.63	*0.60–0.67*
COPD	29.7	36.7	0.85	*0.79–1.92*
dementia	57.7	74.6	1.96	*1.71–2.25*
other	25.0	33.5	0.80	*0.77–0.82*
Multimorbidity (*p* < 0.001)				
*no (ref.)*	31.1	38.3	*1.00*	
yes	30.5	34.4	1.01	*0.98–1.05*
unknown (no hospital stay)	23.8	30.9	0.75	*0.73–0.78*
ICU during last stay (*p* < 0.001)				
*no (ref.)*	28.3	*34.8*	*1.00*	
1*–*48 h	19.0	27.9	0.69	*0.66–0.73*
more than 48 h	29.8	31.3	1.07	*1.01–1.13*
Educational level (*p* < 0.001)				
*medium (ref.)*	28.3	33.2	*1.00*	
low	26.4	32.5	0.96	*0.93–0.99*
high	29.9	37.8	1.04	*0.99–1.09*
unknown	27.6	37.4	1.03	*0.99–1.08*
House or flat owner (*p* < 0.001)				
*tenant (ref.)*	28.3	34.6	*1.00*	
owner-occupier	26.8	33.4	0.93	*0.90–0.96*
Room category (*p* < 0.01)				
*regular (ref.)*	26.9	*35.0*	*1.00*	
semi-private/private	29.7	31.3	1.08	*1.04–1.11*
Marital status (*p* < 0.001)				
*married (ref.)*	28.5	33.4	*1.00*	
never married	29.4	40.7	1.04	*0.98–1.09*
widowed	25.1	32.5	1.00	*0.96–1.04*
divorced	32.5	37.8	1.10	*1.03–1.16*
Nationality (*p* < 0.001)				
*Swiss (ref.)*	27.3	33.8	*1.00*	
foreign	33.0	38.2	1.09	*1.03–1.16*
Nursing home bed density^a^ (*p* > 0.001)			0.97	*0.97–0.98*

*IRR* incidence rate ratios, 95 % CI = 95 % confidence interval, *p*-values from likelihood ratio tests

^a^ = average number of nursing home beds per 100 habitants aged 65 years and older (per 106 regions)

Data source: Swiss Federal Statistical Office, MedStat, SNC

**Table 4:** Results for the French speaking part of Switzerland (*N* = 9,745)

	Length of stay (days)		IRR	*95 % CI*
	Mean	SD		
Age (at time of death) (*p* < 0.001)			0.99	*0.99–0.99*
Sex (*p* < 0.05)				
men	41.6	46.5	*1.00*	
women	42.6	46.5	1.01	*0.97–1.06*
Cause of death (*p* < 0.001)				
*cancer (ref.)*	48.1	42.1	*1.00*	
coronary heart disease	30.7	43.4	0.67	*0.62–0.73*
stroke	32.8	46.6	0.70	*0.65–0.77*
COPD	49.8	58.1	0.96	*0.85–1.09*
dementia	70.0	72.3	1.54	*1.31–1.82*
other	38.5	47.5	0.84	*0.80–0.89*
Multimorbidity (*p* < 0.001)				
*no (ref.)*	40.6	43.0	*1.00*	
yes	48.0	51.0	1.22	*1.15–1.30*
unknown (no hospital stay)	36.8	42.6	0.91	*0.86–0.96*
ICU during last stay (*p* < 0.001)				
*no (ref.)*	44.5	*47.7*	*1.00*	
1*–*48 h	23.8	35.7	0.54	*0.49–0.58*
more than 48 h	33.9	37.5	0.76	*0.71–0.82*
Educational level (*p* < 0.001)				
*medium (ref.)*	40.5	43.6	*1.00*	
low	41.2	48.9	1.02	*0.96–1.07*
high	43.9	48.0	1.09	*1.02–1.17*
unknown	44.7	50.4	1.09	*1.03–1.16*
House or flat owner (*p* < 0.001)				
*tenant (ref.)*	44.1	49.2	*1.00*	
owner-occupier	39.2	42.2	0.92	*0.88–0.97*
Room category (*p* < 0.01)				
*regular (ref.)*	42.9	*47.6*	*1.00*	
semi-private/private	35.0	34.5	0.83	*0.77–0.89*
Marital status (*p* < 0.001)				
*married (ref.)*	40.9	43.9	*1.00*	
never married	41.8	48.5	1.02	*0.94–1.11*
widowed	42.0	47.5	1.08	*1.02–1.14*
divorced	50.0	55.3	1.15	*1.06–1.25*
Nationality (*p* < 0.001)				
*Swiss (ref.)*	41.1	45.5	*1.00*	
foreign	49.9	53.5	1.11	*1.04–1.19*
Nursing home bed density^a^ (*p* > 0.001)			0.89	*0.88–0.91*

*IRR* incidence rate ratios, 95 % CI = 95 % confidence interval, *p*-values from likelihood ratio tests

^a^ = average number of nursing home beds per 100 habitants aged 65 years and older (per 106 regions)

Data source: Swiss Federal Statistical Office, MedStat, SNC

## Abbreviations

BIC: Bayesian information criterion
COPD: Chronic obstructive pulmonary disease
IRR: Incidence rate ratio
ISCED: International Standard Classification of Education
MedStat: Medical statistics of the Swiss hospitals
SNC: Swiss National Cohort
